# Longitudinal Association between L1 Trabecular Attenuation from Chest Computed Tomography (CT) and Bone Mineral Density from Dual-energy X-ray Absorptiometry (DXA)

**DOI:** 10.2174/1573405619666230213122733

**Published:** 2023-05-31

**Authors:** Jiyun Lim, Eunsun Oh, Suyeon Park, Hyun-joo Kim, Young Cheol Yoon, Boda Nam, Eun Ji Lee, Jiyoung Hwang, Jewon Jeong, Yun-Woo Chang

**Affiliations:** 1 Department of Radiology, Soonchunhyang University Seoul Hospital, Seoul, Korea;; 2 Department of Biostatistics, Soonchunhyang University Seoul Hospital, Seoul, Korea;; 3 Department of Radiology, Samsung Medical Center, Sungkyunkwan University School of Medicine, Seoul, Korea

**Keywords:** T-score, vertebral trabecular attenuation, Chest Computed Tomography (CT), Dual-energy X-ray absorptiometry (DXA), breast cancer, ROI

## Abstract

**Background:**

Many studies have shown that vertebral trabecular attenuation measured on CT scan corresponds well to DXA results for bone mineral density. These studies were based on cross-sectional data. Hence, there were limitations in explaining the constantly changing vertebral trabecular attenuation from CT and T-score from DXA over time.

**Objective:**

This study aimed to determine the longitudinal association between the vertebral trabecular attenuation measured on computed tomography (CT) and the T-score measured by dual-energy X-ray absorptiometry (DXA).

**Methods:**

We performed a database search for 333 patients who underwent surgery for breast cancer, preoperative treatment, and at least one follow-up chest CT and DXA from January, 2013 through May, 2021. One musculoskeletal radiologist measured the mean vertebral trabecular attenuation of lumbar vertebra 1(L1) on axial unenhanced images at the pedicle level by manually placing the region of interest (ROI). DXA of the lumbar spine was performed, and the lowest T-score of the lumbar spine was used for the analysis. We evaluated the association between L1 trabecular attenuation from chest CT and T-score from DXA over time using the generalized estimating equations (GEE) model to analyze longitudinal corrected data.

**Results:**

A total of 150 women (mean age, 52.4 ± 11.0 years) were included. There was a statistically significant association between L1 trabecular attenuation from chest CT and T-score from DXA in the unadjusted model (*p* < 0.001) and adjusted model (*p* < 0.001). T-score value increased by 0.172 (95% confidence interval (CI): 0.145-0.200, *p* < 0.001) per 10 unit (HU) of L1 trabecular attenuation at time = 0 in unadjusted model and by 0.173 (95% CI: 0.143-0.203, *p* < 0.001) in all adjusted model.

**Conclusion:**

We demonstrated that L1 attenuation from chest CT images was longitudinally associated with T-score from DXA, and the degree of association appeared to be decreased over time in breast cancer patients regardless of their medical condition.

## 1 INTRODUCTION

Osteoporosis is a skeletal disorder characterized by loss of bone strength and causes fractures in the skeletal system [1]. Osteoporotic fractures are a concerning cause of pain, disability, poor quality of life, and increased rates of morbidity and mortality [2, 3]. Therefore, many countries have a great interest in addressing osteoporosis to improve public health [4]. Dual-energy X-ray absorptiometry (DXA) is the most widely used screening tool for diagnosing osteoporosis due to its low level of radiation exposure, intermediate cost, reproducibility, noninvasive nature, good precision, and validation in numerous clinical trials [5, 6]. However, DXA measurements may vary depending on the machine used, different dual-energy methods, different detectors, calibration differences, and different reference standards [6]. Results of DXA may also differ due to aortic calcification, vertebral compression fracture, osteoarthritis, positioning, and anatomical variation in the population [6].

Recently, many studies have been conducted on opportunistic osteoporosis screening [7-11]. Opportunistic osteoporosis screening is the concept that computed tomography scans performed for other clinical indications can be used to estimate bone mineral density in the general population. The vertebral trabecular (non-cortical) attenuation (or density) expressed by Hounsfield units (HU) can be derived from computed tomography (CT) [8]. Previous studies have shown that vertebral trabecular attenuation measured on CT scan corresponds well to DXA results for bone mineral density [8, 11]. However, these previous studies were based on cross-sectional data; hence, there were limitations in explaining the constantly changing vertebral trabecular attenuation from CT and T-score from DXA over time.

Therefore, the objective of this study was to determine the association between vertebral trabecular attenuation measured on CT and T-score measured by DXA over time through longitudinal data.

## 2 MATERIALS AND METHODS

### 2.1 Patients

This retrospective study was approved by the Institutional Review Board (IRB) at the Soonchunhyang University Seoul Hospital. The need for informed consent was waived (IRB no. 2020-03-021-002). Since it was difficult to measure the gradual bone loss due to aging in a short period of time in the general population, we studied breast cancer patients who were known to experience rapid bone loss in a short period of time. We retrospectively performed a search of the computerized hospital information system and radiology information system for 333 patients who underwent surgery for breast cancer, preoperative treatment, and at least one follow-up chest CT and DXA from January, 2013 through May, 2021. We excluded 182 patients with unavailable lumbar vertebra 1 (L1) (n = 100), abnormal lesions on L1, such as vertebral hemangioma, renal osteodystrophy, postpartum osteoporosis, compression fracture (n = 5), bilateral or recurred breast cancer (n = 33), double primary cancer (n = 28), and distant metastasis (n = 17). A total of 150 patients were finally included. The medical records of enrolled patients were reviewed. Potential covariates of bone mineral density during the study, including age at initial CT, body mass index, aromatase inhibitors, chemotherapy, radiation therapy, tamoxifen, zoladex, osteoporosis medication, and menopause, were also collected.

### 2.2 Image Acquisition

All chest CT images were acquired using two CT scans (SOMATOM Definition Edge, Siemens Medical Solutions, Erlangen, Germany; and Discovery CT750 HD, GE Healthcare, Chicago, IL, USA). Automatic tube-current modulation protocols were adopted when using both CT scans. Our study patients were examined at 120 kVp. The slice thickness was 3.0mm. With each patient in a supine position, both unenhanced and contrast-enhanced CT images were obtained from lung bases through the thoracic inlet level during a single inspiratory breath-hold. Intravenous injection of a contrast medium, either Iomeron 300 (Braco, Milan, Italy) or Optiray 320 (Reyon Pharmaceutical, Seoul, Korea), was injected at 1.5 mL/kg body weight with an infusion rate of 3 mL/sec using a power injector.

DXA of the lumbar spine and proximal femur for BMD assessment was performed with a Lunar Prodigy densitometer (GE Healthcare, Waukesha, Wisconsin, USA) and a Horizon densitometer (HOLOGIC, Marlborough, MA, USA) using standard techniques in accordance with the 2019 International Society for Clinical Densitometry official positions [12]. The measured BMD was transformed into T-score using a manufacturer-provided Korean population reference value. The lowest T-score of lumbar spines was used for the analysis. All DXA images were interpreted blindly to the results of CT attenuation. There was a one-week interval between chest CT and DXA.

### 2.3 Image Analysis

**Fig. (1):**
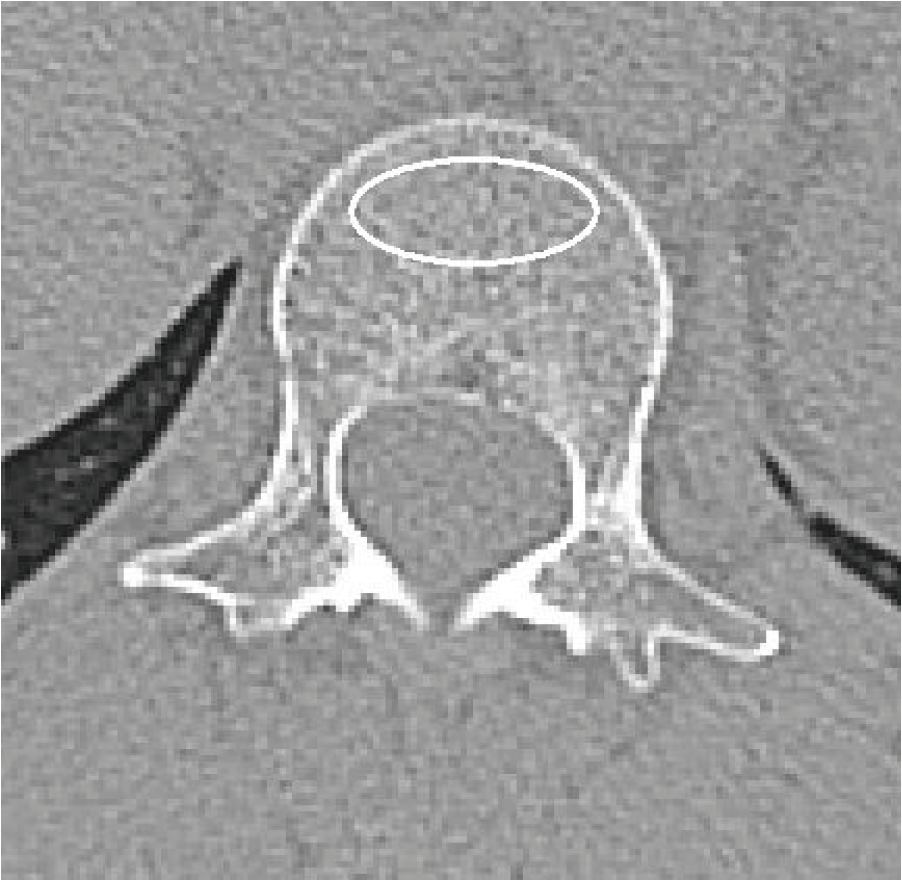
CT-based vertebral trabecular attenuation of L1 for assessment of bone mineral density. The average trabecular attenuation of L1 was assessed by manually placing a region of interest (ROI) in the anterior aspect of the L1 trabecular space.

One musculoskeletal radiologist with 5 years of experience following fellowship training measured the average trabecular attenuation of L1 on axial unenhanced images at the pedicle level blinded to DXA results and medical records. The average trabecular vertebral attenuation of L1 was assessed by manually placing an individual oval-shaped ROI in the anterior aspect of the L1 trabecular space at the pedicle level, excluding cortical bone and focal lytic or sclerotic lesions (Fig. **1**) [9, 13]. Image assessment and measurements were all performed using our institutional PACS.

### 2.4 Statistical Analysis

Statistical analyses were performed in SPSS (version 22; IBM Corp., Armonk, NY). We evaluated the association between L1 trabecular attenuation from chest CT and T-score from DXA over time using the generalized estimating equations (GEE) model to analyze longitudinal correlated data. Model 1 was unadjusted, and Model 2 was adjusted for age at initial CT, body mass index, aromatase inhibitors, chemotherapy, radiation therapy, tamoxifen, zoladex, osteoporosis medication, and menopause. To further assess the differences in the longitudinal association between L1 trabecular attenuation and T-score by age, patients were divided into those under 55 and over 55 years of age. A *p*-value of less than 0.05 was statistically significant.

## 3 RESULTS

**Fig. (2):**
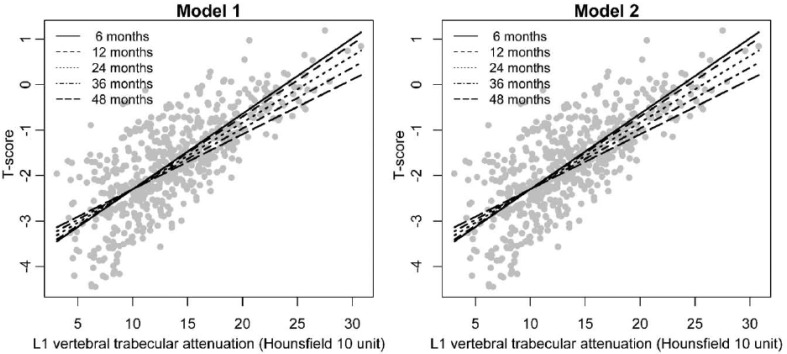
Scatter plot represents the association between L1 trabecular attenuation and T-score at different follow-up times in unadjusted (Model 1) and adjusted models (Model 2). Regression lines are calculated using generalized estimating equation models to accommodate repeated measures from the same patients. The slope of correlation shows a tendency to decrease slightly over time.

The baseline characteristics of the participants are summarized in Table **1**. A total of 150 patients were included in our study. All patients were women, and the initial mean age was 52.4 ± 11.1 years. Among 150 patients, 7 patients had 2 scans, 35 patients had 3 scans, 62 patients had 4 scans, and 46 patients had 5 scans since enrollment. As shown in Table **2**, Model 1 is the unadjusted model, and Model 2 is the adjusted model for chemotherapy, radiation therapy, medication (tamoxifen, aromatase inhibitor, zoladex, bisphosphonate), menopause state, and body mass index. This is because the target group of this study is breast cancer patients, and bone mineral density may be affected by these various factors. Table **2** shows a statistically significant association between L1 trabecular attenuation from chest CT and T-score from DXA in both Models 1 and 2. The T-score value increased by 0.172 (95% confidence interval (CI): 0.145-0.200, *p* < 0.001) per 10 unit (HU) of L1 trabecular attenuation at time = 0 in model 1 and by 0.173 (95% CI: 0.143-0.203, *p* < 0.001) in model 2. In the longitudinal analyses, a statistically significant interaction was found for L1 trabecular attenuation and follow-up time in both Models 1 and 2 (beta = -0.001, 95% CI: -0.002-0.000, *p* = 0.009 for model 1; beta=-0.001, 95% CI: -0.002-0.000, *p* = 0.002 for model 2), indicating that the association of L1 trabecular attenuation and T-score appeared to be changed over time. The degree of correlation showed a tendency to decrease slightly over passing time because of significant interaction between L1 trabecular attenuation and follow-up time (Fig. **2**).

In order to further dissect this longitudinal correlation by age, the population was classified under 55 and over 55 years of age. For Model 2, there was a significant decrease by -0.001 (95% CI: -0.002-0.000, *p* = 0.025) in the group under 55 years of age and an increase by 0.000 (95% CI: -0.002-0.001, *p* = 0.619) in the group over 55 years of age; however, it was not statistically significant (Table **S1**).

## 4 DISCUSSION

In this longitudinal retrospective study, we demonstrated a significant correlation between L1 trabecular vertebral attenuation extracted from CT and T-score from DXA over time. The association of L1 trabecular attenuation and T-score appeared to be changed over time.

There are many studies on the opportunistic use of CT images acquired for other clinical indications for osteoporosis screening and bone density evaluation [8, 9, 14]. CT-based vertebral trabecular attenuation can be measured by simply drawing ROI. This approach showed excellent interobserver agreement in previous studies [13, 15, 16]. Jang *et al*. demonstrated a normative range of L1 trabecular attenuation across all adult ages in constructed CT images [9]. Geroty *et al*. demonstrated that L1 trabecular attenuation could be a reliable indicator of bone mineral density for opportunistic osteoporosis screening [15]. Many studies have proposed optimal thresholds for vertebral trabecular attenuation to determine osteoporosis [8, 10, 17]. Thus, vertebral trabecular attenuation is considered to assess bone mineral density.

The association between trabecular attenuation and T-score from DXA has previously been described in cross-sectional studies. However, this study is the first to find a statistically significant association in longitudinal follow-up. Pickhardt *et al*. and Cohen *et al*. showed that vertebral trabecular attenuation correlated with T-scores from DXA in cross-sectional studies [8, 17]. These previous studies and our findings suggested the capability of providing additional information related to bone mineral density from routinely repeated CT scans without any additional radiation exposure, imaging time, and cost. From this longitudinal correlation between trabecular attenuation from CT and T-score from DXA, trabecular attenuation can be used continuously for evaluating bone mineral density in the case of patients inevitably having to take repeated CT scans.

Since it was difficult for the general population to measure the gradual bone loss caused by aging, a study was conducted on breast cancer patients who suffered from rapid bone loss in a short time [18-20]. Ramin *et al*. demonstrated that the incidence of bone loss was significantly higher in breast cancer survivors treated with chemotherapy or hormone therapy [19]. We tested the longitudinal association between L1 trabecular attenuation and T-score in the unadjusted model (Table **2**, Model 1) and fully adjusted model, including chemotherapy, radiation therapy, medication, menopause, and body mass index (Table **2**, Model 2). We found that L1 trabecular attenuation was positively associated with T-score in both models. In addition, aging is one of the most common causes of bone loss in women, and we considered the interaction between follow-up time with L1 trabecular attenuation and T-score. In our study, a statistical interaction was reported between L1 trabecular attenuation and T-score with follow-up time, indicating that the association of L1 trabecular attenuation and T-score appeared to be changed over time. Our finding suggested that the degree of correlation tended to decrease slightly over time. This was because degenerative scoliosis and osteophyte formation caused errors in DXA results with age. We suggested that vertebral trabecular attenuation should be supplemented with DXA in the degenerative and scoliotic spine.

People with chronic diseases, such as cystic fibrosis or diabetes mellitus and long-term medication with inflammatory bowel disease, organ transplant recipients, or cancer survivors commonly underwent follow-up DXA to monitor bone mineral density in addition to repeated diagnostic CT scans [21-25]. Our finding supported that these patients could evaluate the change of bone mineral density with regularly performed diagnostic CT images without additional radiation exposure or cost.

Our study has several limitations. First, it was a retrospective, single-center investigation with a small sample size of women who had breast cancer; therefore, it might have been influenced by verification and selection bias. Further studies are required to assess longitudinal association in other larger patient cohorts. Second, there might be scan-related factors that could influence bone attenuation measurement since we used two different DXA scans and two different CT scans [26]. Third, we excluded many chest CT images that did not include L1 on unenhanced images (n = 100). This is because our hospital tends to narrow the scan range when taking unenhanced images to reduce radiation exposure.

## CONCLUSION

In conclusion, L1 trabecular attenuation from CT images was found to be longitudinally associated with T-score from DXA, and the degree of association appeared to be decreased over time in breast cancer patients regardless of their medical condition.

## Figures and Tables

**Table 1:** Baseline characteristics of enrolled patients.

**Variables**	**Number**
Age (years) ^i^	52.4 ± 11.0 (28 - 81)
Female	150
Weight (kg) ^i^	58.5 ± 9.3
Height (kg) ^i^	157.2 ± 5.6
Body mass index ^i^	23.8 ± 3.9
Follow-up period (months) ^i^	23.8 ± 18.4 (10 - 71)
Surgery, n (%)	-
Mastectomy	54 (36%)
Breast conserving surgery	96 (64%)
Pathology, n (%)	-
Invasive ductal carcinoma	137 (91.3%)
Ductal carcinoma *in situ*	4 (2.7%)
Invasive lobular carcinoma	8 (5.3%)
Metaplastic carcinoma	1 (0.7%)
Chemotherapy, n (%)	88 (58.7%)
Radiation therapy, n (%)	99 (66%)
Aromatase inhibitor use, n (%)	83 (55.3%)
Tamoxifen use, n (%)	63 (42%)
Zoladex use, n (%)	24 (16%)
Osteoporosis medication, n (%)	25 (16.7%)
Menopause status, n (%)	80 (53.3%)
Initial L1 trabecular attenuation ^i^	157.5 ± 53.4
Initial T-score ^i^	-1.3 ± 1.4

**Note:** CT = computed tomography, DXA = dual-energy X-ray absorptiometry.

i. Data are mean ± standard deviation.

**Table 2:** Generalized estimating equation for T-score from dual-energy X-ray absorptiometry (DXA).

**Variable**	**Model 1**				**Model 2**			
	**Beta**	**SE**	**95% CI**	**P value**	**Beta**	**SE**	**95% CI**	***P-*value**
L1 trabecular attenuation	0.172	0.014	0.145, 0.200	<0.001	0.173	0.015	0.143, 0.203	<0.001
Follow-up time (months)	0.011	0.006	-0.001, 0.022	0.072	0.010	0.005	0.001, 0.019	0.022
Follow- up time *L1 trabecular attenuation	-0.001	0.0004	-0.002, 0.000	0.009	-0.001	0.0003	-0.002, 0.000	0.002

**Note:** Model 1 unadjusted, model 2 adjusted for chemotherapy, radiation therapy, medication (tamoxifen, aromatase inhibitor, zoladex, bisphosphonate), menopause state, body mass index. Data are difference in T-score per 10 unit (Hounsfield unit) of L1 trabecular attenuation.

## Data Availability

Not applicable.

## LIST OF ABBREVIATIONS

CI: Confidence Interval
CT: Computed Tomography
GEE: Generalized Estimating Equations
ROI: Region of Interest
