# Supplementary material for: Longitudinal Association between L1 Trabecular Attenuation from Chest Computed Tomography (CT) and Bone Mineral Density from Dual-energy X-ray Absorptiometry (DXA)
Source: Curr Med Imaging. 2023 May 31;19(12):1372–7. doi: 10.2174/1573405619666230213122733 (PMC10353069; doi:10.2174/1573405619666230213122733)
Supplement: Supplementary file 1 [file CMIM-19-1372_SD1.pdf]

Supplementary Material

Longitudinal Association between L1 Trabecular Attenuation from Chest Computed Tomography (CT) and Bone Mineral Density from Dual-energy X-ray Absorptiometry (DXA)

Jiyun Lim[1], Eunsun Oh[1,*], Suyeon Park[2], Hyun-joo Kim[1], Young Cheol Yoon[3], Boda Nam[1], Eun Ji Lee[1], Jiyoung Hwang[1], Jewon Jeong[1] and Yun-Woo Chang[1]

[1]Department of Radiology, Soonchunhyang University Seoul Hospital, Seoul, Korea; [2]Department of Biostatistics, Soonchunhyang University Seoul Hospital, Seoul, Korea; [3]Department of Radiology, Samsung Medical Center, Sungkyunkwan University School of Medicine, Seoul, Korea

Supplement Table 1. Generalized estimating equation for T-score from Dual-energy X-ray absorptiometry (DXA) by age.

Variable	Model 1				Model 2			
	beta	SE	95% CI	P value	beta	SE	95% CI	P value
L1 trabecular attenuation	0.193	0.018	0.157, 0.228	<0.001	0.175	0.016	0.143, 0.207	<0.001
Follow-up time (months)	0.007	0.009	-0.011, 0.024	0.462	0.008	0.007	-0.005, 0.021	0.237
Age (over 55 years)	0.333	0.240	-0.138, 0.804	0.166	0.366	0.193	-0.013, 0.745	0.058
Age (under 55 years) * Follow-up time * L1 trabecular attenuation	-0.001	0.0005	-0.002, 0.000	0.121	-0.001	0.0004	-0.002, 0.000	0.025
Age (over 55 years) * Follow-up time * L1 trabecular attenuation	-0.0001	0.001	-0.002, 0.002	0.960	0.000	0.0008	-0.002, 0.001	0.619

Model 1 unadjusted, model 2 adjusted for chemotherapy, radiation therapy, medication (tamoxifen, aromatase inhibitor, zoladex, bisphosphonate), menopause state, body mass index. Data are difference in T-score per 10 unit (Hounsfield unit) of L1 trabecular attenuation.
